# Derivation of Corneal Endothelial Cell-Like Cells from Rat Neural Crest Cells In Vitro

**DOI:** 10.1371/journal.pone.0042378

**Published:** 2012-07-31

**Authors:** Chengqun Ju, Kai Zhang, Xinyi Wu

**Affiliations:** 1 Department of Ophthalmology, Qilu Hospital, Shandong University, Jinan, People’s Republic of China; 2 The Key Laboratory of Cardiovascular Remodeling and Function Research, Chinese Ministry of Education and Chinese Ministry of Health, Qilu Hospital, Shandong University, Jinan, People’s Republic of China; University of Minnesota Medical School, United States of America

## Abstract

The aim of this study was to investigate the feasibility of inducing rat neural crest cells (NCC) to differentiate to functional corneal endothelial cell (CEC)-like cells in vitro. Rat NCC were induced with adult CEC-derived conditioned medium. Immunofluorescence, flow cytometry and real time RT-PCR assay were used to detect expression of the corneal endothelium differentiation marker N-cadherin and transcription factors FoxC1 and Pitx2. CFDA SE-labeled CEC-like cells were transplanted to the corneal endothelium of a rat corneal endothelium deficiency model, and an eye-down position was maintained for 24 hours to allow cell attachment. The animals were observed for as long as 2 months after surgery and underwent clinical and histological examination. Spindle-like NCC turned to polygonal CEC-like after induction and expressed N-cadherin, FoxC1, Pitx2, zonula occludens-1 and sodium-potassium pump Na^+^/K^+^ ATPase. The corneas of the experimental group were much clearer than those of the control group and the mean corneal thickness in the experimental group was significantly less than in the control group7, 14, 21 and 28 days after surgery. Confocal microscopy through focusing and histological analysis confirmed that green fluorescence-positive CEC-like cells formed a monolayer covering the Descemet’s membrane in the experimental group. In conclusion, CEC-like cells derived from NCCs displayed characters of native CEC, and the induction protocol provides guidance for future human CEC induction from NCC.

## Introduction

Tissue engineering of the cornea has been presented as a promising opportunity for overcoming the limitations of conventional corneal replacement, such as shortage of healthy donor corneas and possible allograft rejection [1]–[4]. One of the important elements about artificial cornea construction is the source of cells which can be seeded on or in the scaffolds. However, the acquisition of large amount of corneal endothelial cells (CEC) by in vitro cultivation is confronted with major challenges. First, the number of suitable donor corneas for cell isolation is quite limited, and second, unlike corneal epithelial and stroma cells, CEC have extremely low mitotic activity under standard cell culture condition [5]. Thus finding other cell types which could substitute for CEC becomes an urgent issue at the moment.

It is known that both human and rodent CEC originate from neural crest cells (NCC) [6]–[11]. The neural crest is a transient cell population in the early stage of embryonic development, which gives rise to many kinds of tissues and cell types. At present NCC can not only be acquired from fetus, but also from adult skin, hair follicle and even embryonic stem cell-induction in both human and rodent [12]–[17]. Direct precursor of CEC, multiple differentiation ability in vitro and extended sources make NCC a candidate cell source for CEC induction.

Research on inductive differentiation of CEC is quite a new area, and no experimental protocol has been established yet. We took advantage of former induction protocols in other cell types and known factors that could influence the fate of cells to find a protocol which might be used to realize the conversion of NCC to CEC. We showed in this study that conditioned medium (CM) derived from CEC combining with serum could induce rat NCC to differentiate into CEC-like cells, and we also tested the effect of CEC-like cell transplantation in a rat model of corneal endothelium deficiency.

## Results

### Cultivation and Identification of Rat NCC

**Figure 1:**
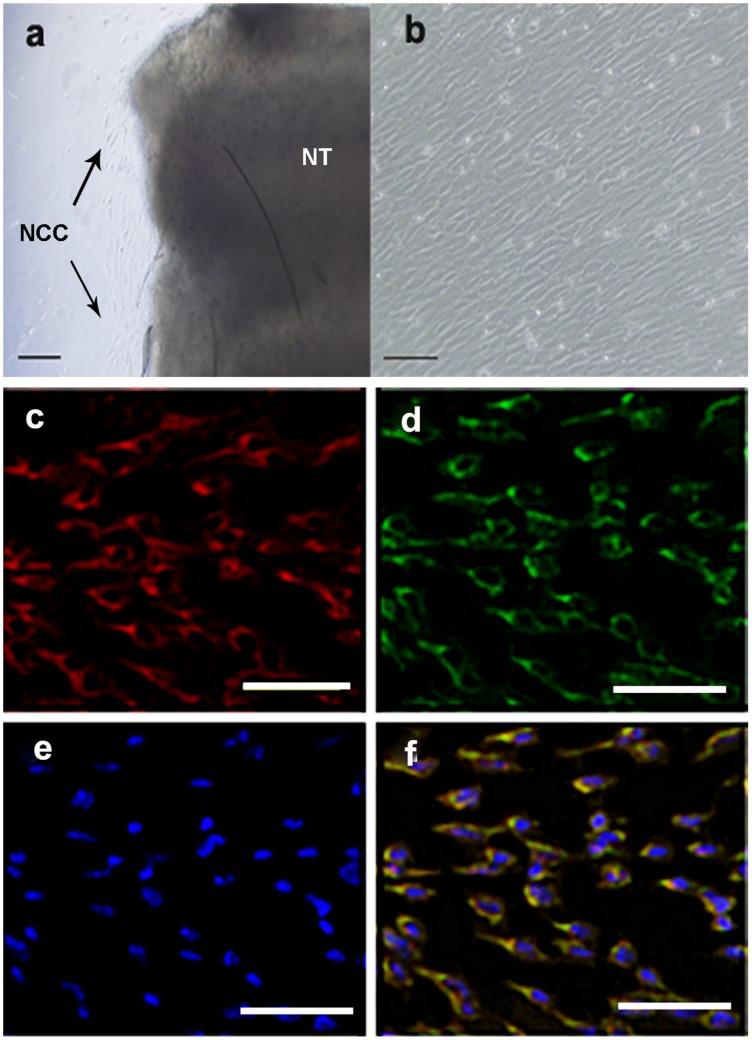
Primary culture and identification of rat neural crest cells. (a) Spindle-like cells migrated from the neural tube of a rat embryo 24 hours after culture. (b) The cells reached confluence 7 days after culture. Spindle-shaped cells simultaneously expressed P75 (c) and HNK-1(d). (e) DAPI staining of cell nuclei. (f) Overlay image of c, d and e. NT: neural tube; NCC: neural crest cell. Scale bar: 100 µm.

Rat NCC quickly migrated from the neural tube and displayed an elongated spindle-like shape. The cells showed a good growth rate and reached confluence around the seventh day after the neural tube was removed (Fig. 1a and b). Low-affinity neurotrophin receptor P75 and the HNK-1 epitope have been used as general markers for neural crest cells, thus we co-immunostained the cells for both P75 and HNK-1 [18]–[21]. The results showed that P75^+^ cells also expressed HNK-1, which confirmed their identity as NCC (Fig. 1c, d, e and f).

### Characterization of Rat NCC Differentiation

**Figure 2:**
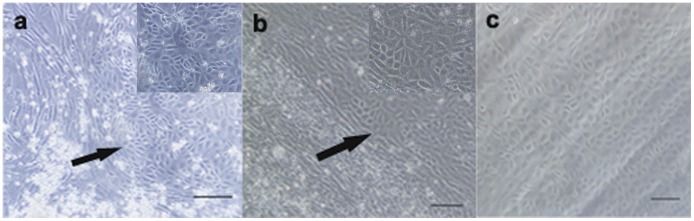
Morphological change of NCC after induction. (a) A small part of NCC changed to polygonal shape (arrow) 7 days after induction. (b) On day 14, about half of the cells in one field became polygonal (arrow). (c) Normal rat CEC control. Insets in a and b are higher magnifications of polygonal cells. Scale bars: 100 µm.

Morphological change of rat NCC from spindle shape to polygonal CEC-like shape began on day 7 after being cultured with the differentiation medium (Fig. 2a). The ratio of CM and fresh DMEM/F12 was optimized during preliminary experiments, and we found that the ratio of 3∶1 was better than others (such as 1∶3, 1∶1). Serum was also required for NCC differentiation. In this study we observed that the differentiation medium with 10% FBS could well support proliferation and differentiation of NCC. About 40–50% of NCC changed their morphology to CEC-like shape when cultured up to 14 days. Polygonal cells closely connected to one another which mimicked native CEC cultured in vitro (Fig. 2b and c).

**Figure 3:**
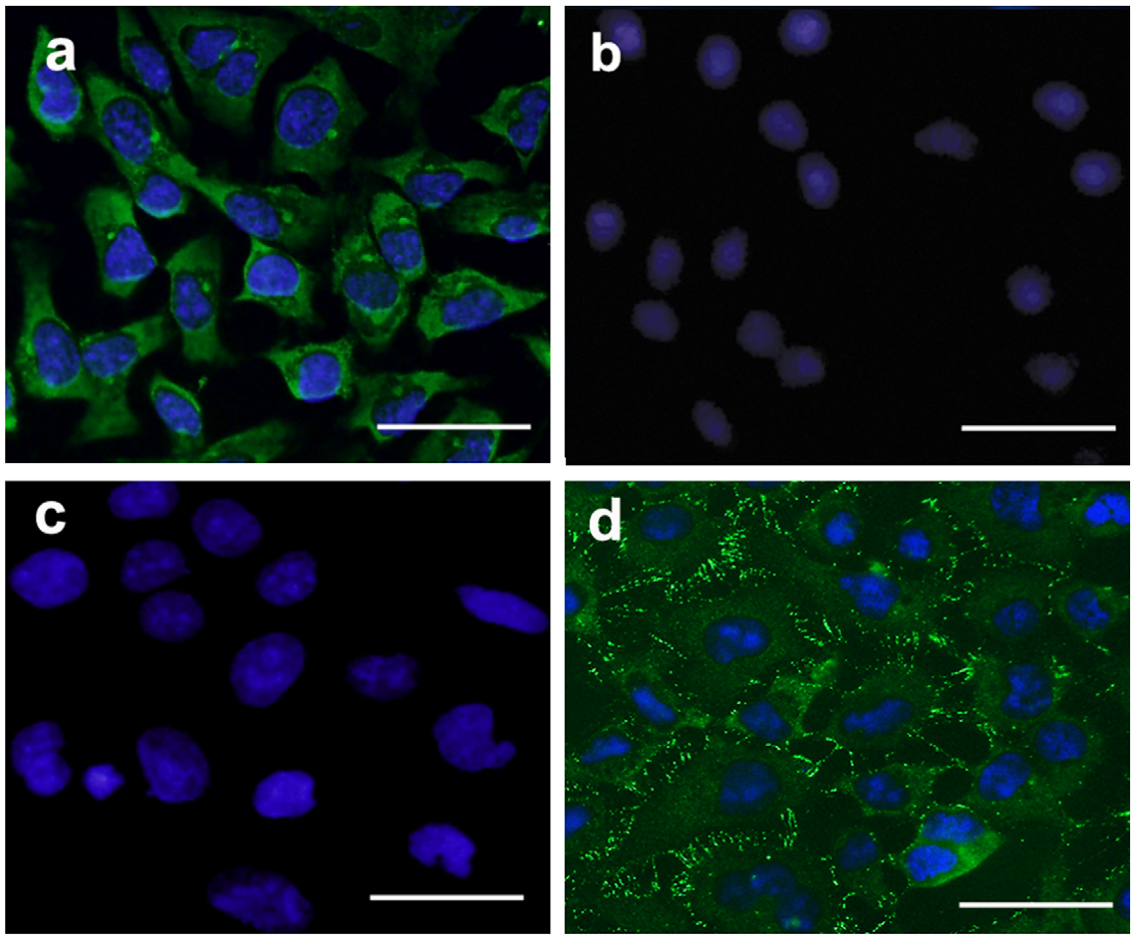
Immunofluorescence detection of N-cadherin. (a) Cells were stained with N-cadherin antibodies after induction, green fluorescence mainly distributed in the plasma and cell membrane. (b) No positive signal was detected among non-induced NCC. (c) No positive signal was detected in negative control. (d) Normal rat CEC were stained with N-cadherin antibodies, green fluorescence was mainly detected at cell boundaries. Scale bars: 50 µm.

It is known that N-cadherin is expressed by developing rodent CEC [22], and therefore it was used as a marker to identify CEC differentiation. As shown in Fig. 3a, expression of N-cadherin can be seen in the cytoplasm of the induced cells. There was no detectable fluorescence among non-induced NCC (Fig. 3b) and negative control groups (primary antibody omitted) (Fig. 3c). Normal rat CEC also express N-cadherin, but its distribution mainly restricted in the membrane at cell boundaries (Fig. 3d).

**Figure 4:**
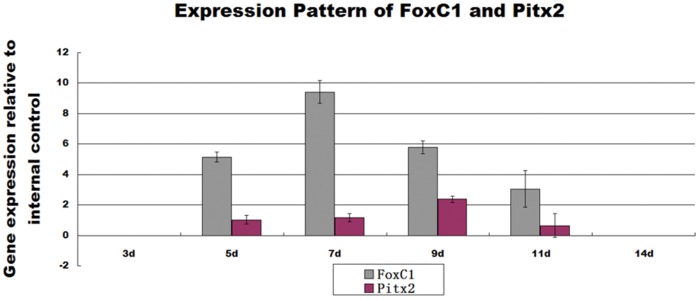
RT-PCR detection of transcription factor genes FoxC1 and Pitx2. Both of these two genes were detected as early as 5 days after induction. The expression level of FoxC1 peaked on day 7, and quickly fell afterwards. Expression of Pitx2 was steady but lower than that of FoxC1, and the peak value appeared around day 9. Error bars  = SD.

Transcription factors FoxC-1 and Pitx-2 both proved to be closely related to CEC development [23], [24], therefore real-time RT-PCR was performed to determine whether expression of these two transcription factors was turned on in the induced NCC. Expression of FoxC-1 peaked on day 7 and then quickly fell. Compared with FoxC1, expression of Pitx2 was lower but at a stable level at the checked time points (Fig. 4). The peak value appeared around day 9, a bit later than that of FoxC1. Non-induced NCC groups showed no amplification of target genes, the same as negative controls (samples omitted) (data not shown).

**Figure 5:**
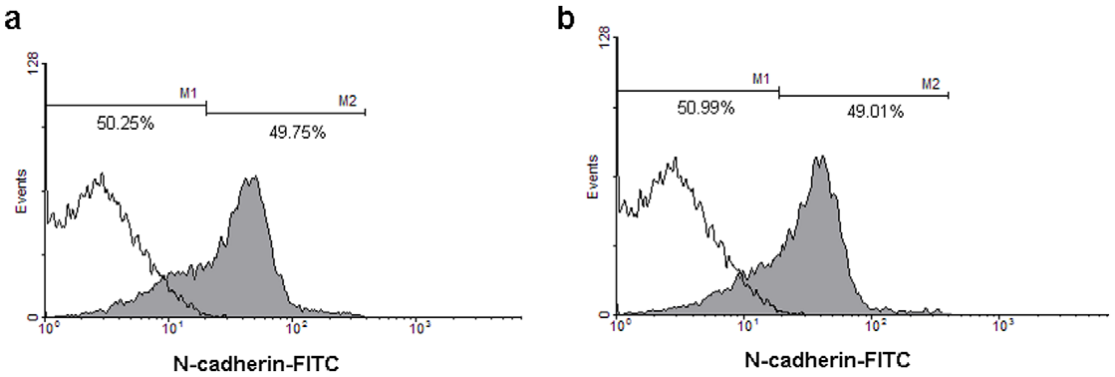
Cell inductivity determined by flow cytometery analysis. (a) The mean proportion of N-cadherin positive cells was about 49.75% 14 days after induction. (b) The mean proportion of N-cadherin positive cells was about 49.01% 20 days after induction. There was no increase in cell inductivity by prolonging the inducing time from 14 days to 20 days. M1: N-cadherin negative cells; M2: N-cadherin positive cells.

One interesting observation was that the percentage of polygonal cells would not increase after 14 days of induction. We prolonged the induction time from 14 days to 20 days, but no increase of N-cadherin-positive cells was observed by flow cytometry analysis (Fig. 5). It seemed that the residual cells “refused” to differentiate under the present system. Those cells remaining N-cadherin negative after 14 days’ induction could not live under the induction condition and gradually detached from the substrate and died.

**Figure 6:**
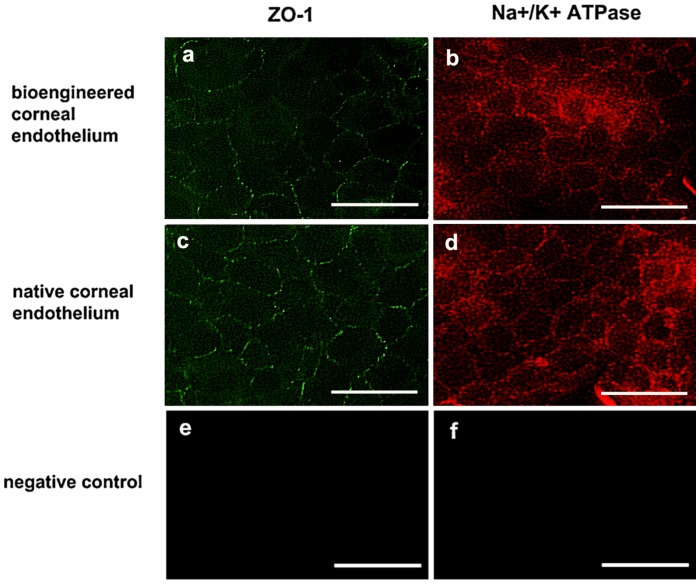
Expression of function-related proteins of the CEC-like cell monolayer on APCM scaffolds. Positive signals for ZO-1 (a) and Na^+^/K^+^ ATPase (b) were detected between cell boundaries of the CEC-like cell monolayer. Expression patterns were similar to normal rat corneal endothelium (c, d), except that fluorescence was weaker for reconstructed corneal endothelium. Primary antibodies were omitted as negative controls (e, f). Scale bars: 50 µm.

Two function-related proteins of CEC, tight junction protein zonula occludens-1 (ZO-1) and sodium-potassium pump Na^+^/K^+^ ATPase were checked by immunofluorescence assay to determine whether the induced cells could form mature corneal endothelium on APCM. APCM scaffolds were generated according to the protocol developed in our lab [25], [26]. CEC-like cells were seeded on the surface of the APCM (3000 cells/mm^2^ in density) and incubated under normal culture conditions for 14 days to promote cell adhesion and connection. As shown in Fig. 6, positive signals for both of ZO-1 and Na^+^/K^+^ ATPase were detected throughout the CEC-like cell membrane (Fig. 6a and b). Such staining pattern was similar to native rat corneal endothelium (Fig. 6c and d), also the fluorescence of native rat CEC seemed more intense than that of CEC-like cells.

**Figure 7:**
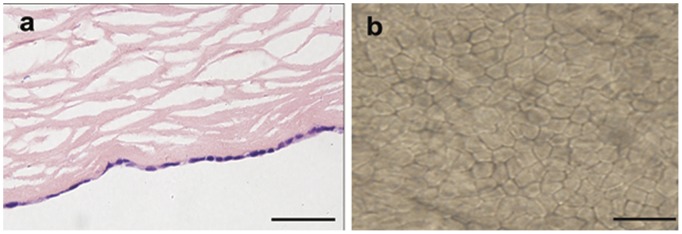
Contact-inhibition analysis of CEC-like cells. (a) HE staining of the reconstructed corneal endothelium showed that CEC-like cells kept a monolayer after 30 days cultivation on APCM in cross section view. (b) Alizarin red S and trypan blue double-staining of the reconstructed endothelium showed the “lake reaction” of cell borders and no multiple layers from plane view. Scale bars: 50 um.

HE staining and alizarin red S-trypan blue double-staining showed that CEC-like cells formed a strict monolayer on APCM in cross section view and plane view, indicating that the cells were contact-inhibited. No tendency was observed to form multiple layers as long as 30 days’ cultivation (Fig. 7).

### Clinical Observations after Surgery

**Figure 8:**
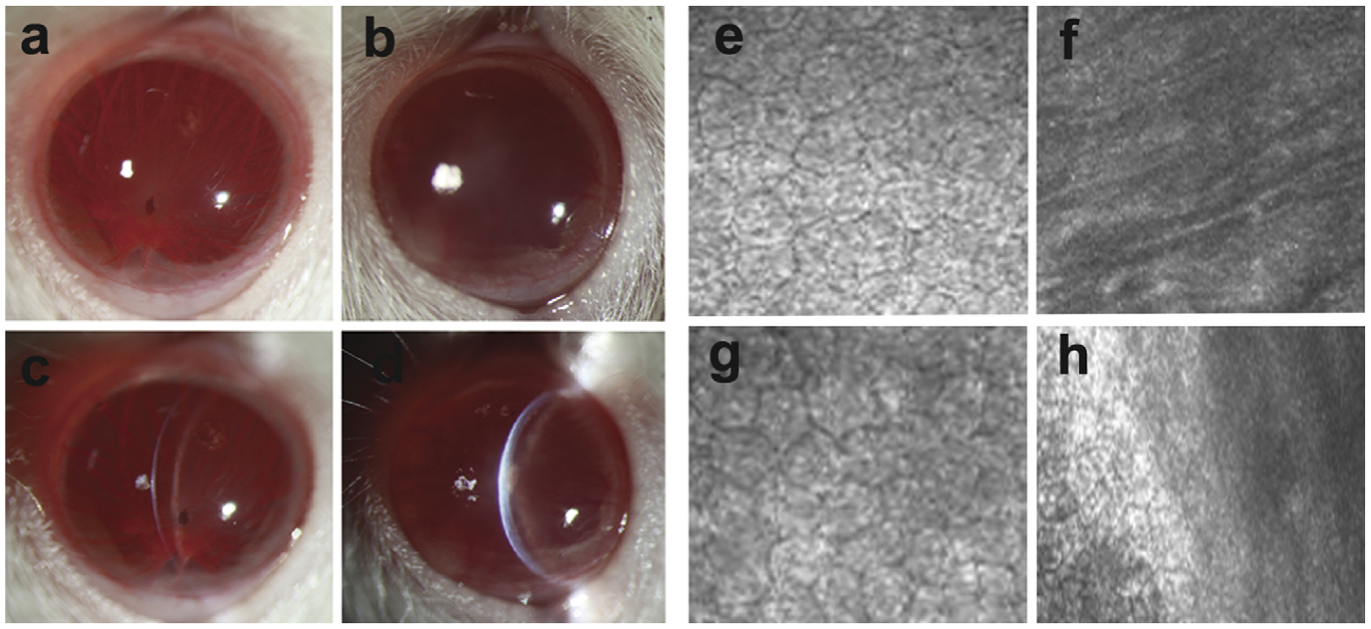
Representative corneal photos after the surgery. Corneas in the experimental group were clear, and the anterior chamber and the iris were clearly visible at 28 days (a). In contrast, corneas in the control group were opaque (b). Corneas in the experimental group were much thinner than that in the control group and the endothelium surface was also smoother under slit observation at 28 days (c and d). Confocal microscopy confirmed the polygonal cell coverage of the Descemet’s membrane in the experimental group at 28 days and 2 months (e and g), while there was only denuded Descemet’s membrane in the control group at 28 days (f), and there was some newborn CEC at the marginal region at 2 months in the control group (h).

**Figure 9:**
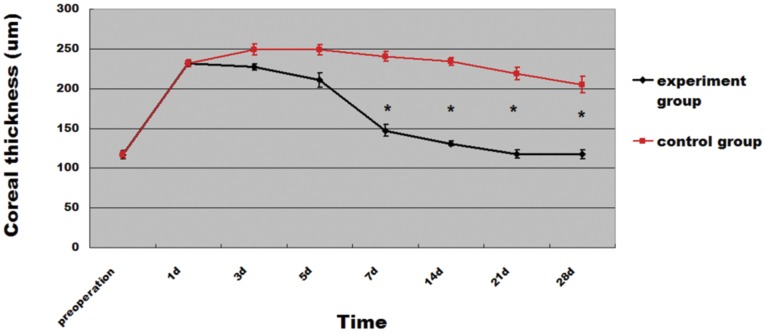
Changes of corneal thickness during clinical observation. Mean corneal thickness of normal rats was about 116±5.27 um. 1 day after cryo-injury, it sharply increased in both experimental and control groups to about two times the normal value. Then it rapidly decreased in the experimental group and was significantly less than the control group on 7, 14, 21 and 28 days after the surgery. Error bars  = SD(*P<0.05).

Pump function of CEC-like cells was investigated in a rat corneal endothelium deficiency model. CEC-like cells were labeled with CFDA SE according to the manufacture’s instruction before the surgery. Photos of the anterior segment showed that corneas were not clear with stromal edema in the control group 28 days after operation (Fig. 8b and d), whereas in the experimental group corneas were clearer and the anterior chamber was clearly visible (Fig. 8a and c). Representative confocal microscope images confirmed full coverage of the endothelium surface by polygonal cells in the experimental group and the cell density was 2872.6±172.7 cells/mm^2^ and 2863.8±174.9 cells/mm^2^ 28 days and 2 months after surgery respectively (Fig. 8e and g). The Descemet’s membrane was denuded in the control group 28 days after surgery (Fig. 8f) and was covered by newborn CEC at some limbus areas 2 months later with a cell density of 31.7±6.9 cells/mm^2^ (Fig. 8h). Corneal thickness is an important parameter for evaluating CEC function. As shown in Fig. 9, the mean corneal thickness of the experimental group (117.2±5.5 µm) was significantly less than that of the control group (205.1±10.4 µm) after 28 days observation (P<0.0001). The corneas were edematous in both the experimental group and control group after the surgery, but the corneal thickness rapidly decreased in the experimental group and the corneas were significantly thinner than in the control group after 14 (130.9±3.1 µm), 21 (117.5±5.3 µm), and 28 (117.2±5.5 µm) days (P<0.05). There was no obvious change of the mean corneal thickness in either the experimental group or control group when it was prolonged to 2 months after operation (data not shown).

### Histological Examination and Evaluation of CEC-like Cells in the Rat Model

**Figure 10:**
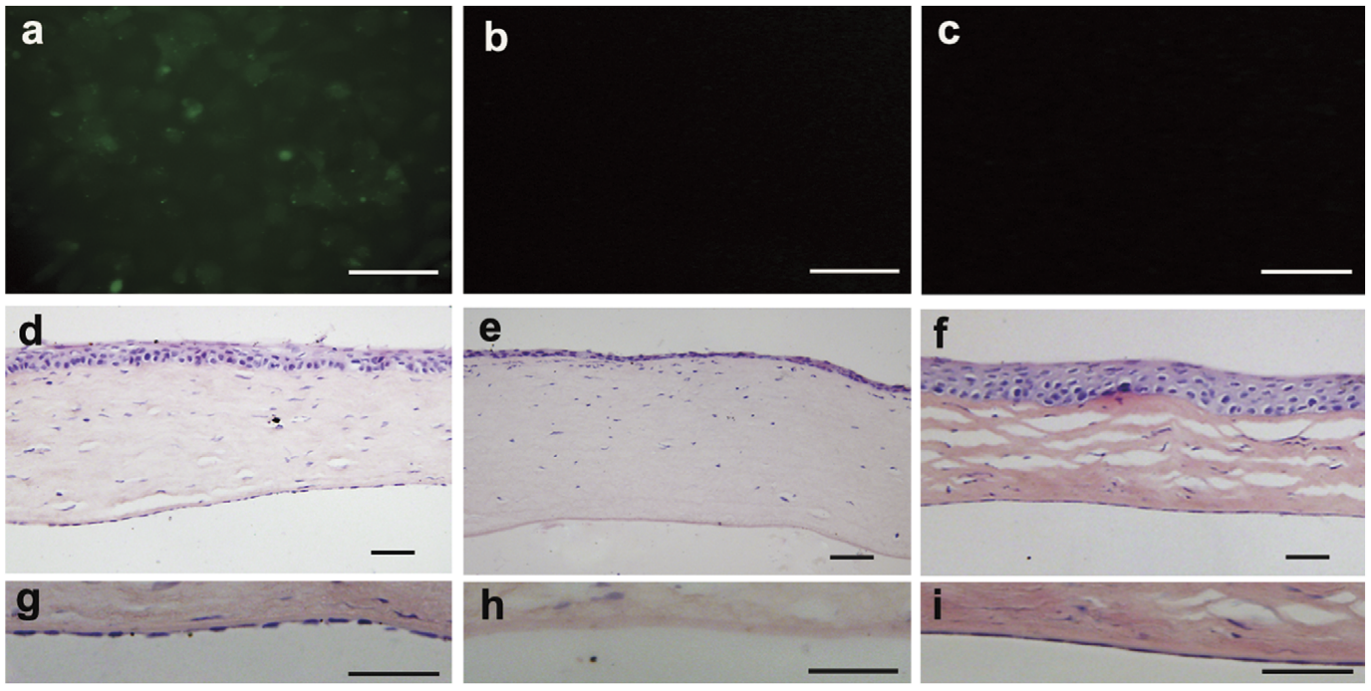
Histological examination of the corneas after the surgery. (a) Cells covering the Descemet’s membrane in the experimental group were CFDA SE-positive under the fluorescence microscope. (b and c) No signal was detected from the corneas of negative control and normal control groups. (d and e) HE-stained cross-section showed similar results that the Descemet’s membrane was covered by a cell monolayer in the experimental group, and no cells were present on Descemet’s membrane in the control group. (f) HE staining of normal rat cornea. (g, h and i) High magnification images of corneal endothelia in d, e and f respectively. Scale bars: 50 µm.

Results of histological examination were consistent with clinical observation. A monolayer of polygonal cells was present on Descemet’s membrane in the experimental group 28 days after operation (Fig. 10a, d and g). These cells were CFDA SE-positive, indicating their origin from CEC-like cells but not host corneal endothelial cells. CEC-like cells attached to Descemet’s membrane in a form similar to normal rat CEC (Fig. 10c, f and i), and cell densities were approximately the same. In contrast, no cells could be seen on Descemet’s membrane in either corneal flat mounts or cross-section HE staining and the corneal stroma edema was prominent in the control group at 28 days (Fig. 10b, e and h).

## Discussion

In the induction experiment we observed that the percentage of CEC-like cells was not directly proportional to the time of induction. After 14 days of induction, the number of CEC-like cells stopped increasing and about half NCC kept their spindle-like shape. The reason for such nonlinear correlation is currently unknown. It is probably due to the fact that only a subpopulation of NCC is composed of progenitor cells which are capable of differentiating into CEC. Another explanation is that the differentiation of NCC has a “set point” and cells that do not receive enough stimuli at the “set point” will miss the chance to differentiate further. Thus looking for new markers of CEC-oriented NCC or optimization induction protocols would be two possible solutions to get as pure CEC-like cells as possible for future research. The proliferative potential was relatively lower once NCC had differentiated to CEC-like cells but still kept good within 3–4 passages. Rodent CEC have stronger proliferation ability compared to human CEC, and maybe that is why rat’s CEC-like cells could be passaged in this study. We assume human cells would probably have more limited reproductive capability, which should be taken into consideration in future research.

Recent research has debated the issue of true differentiation versus cell fusion of stem cells. In this given system, cell fusion is impossible since the induction medium used was totally cell-free. Using CM from CEC has several advantages over other induction methods. First of all, CM collection is simple and convenient; second, using CM could avoid cell fusion problem compared with direct co-culture protocol; and third, it is reported that CM treatment appears more efficient than indirect co-culture protocol, since cell confluence might clog the porous membrane in the top chamber resulting in inefficient leaking of cytokines into the bottom chamber [27]. According to previous studies, CM should be collected when the cells are in their best state and properly stored to save their biological activity. In this study, CM was collected when corneal endothelial cells reached about 70–90% confluence, every 12 h after medium change, and filtered CM was stored at −80°C as suggested. We also observed in our preliminary experiments that NCC poorly thrived and differentiated when they were cultured in medium with less than 10% serum. Serum seems to be necessary for the proliferation and differentiation in this system.

The differentiation medium for NCC induction in this study is a mixture composed of many kinds of unpredictable ingredients, thus it is hard to precisely state which components are effective for CEC-like cell induction. At present, very little is known about the molecular mechanism that regulates the development of corneal endothelium. Previous studies have indicated that ablation of retinoic acid (RA) signaling results in the loss of expression of transcription factor genes of FoxC1 and Pitx2 in NCC and agenesis of corneal endothelium. Current model believes that RA is critically required for anterior segment morphogenesis and NCC are primary direct targets of RA signaling. And activation of FoxC1 and Pitx2 is the transcriptional response within NCC to RA stimulation. RA is an enzymatic metabolite of vitamin A which can be acquired from serum. It is possible that rat CEC might make use of vitamin A existing in fetal bovine serum and secret RA into the CM, and this hypothesis will be further tested in our future experiments. Another potential candidate that is closely related to CEC development is transforming growth factor-β2 (TGF-β2). The existing literatures have revealed that TGF-β2 appears to be the major signaling ligand acting upon NCC during corneal endothelium differentiation. Ablation of TGF-β receptor 2 leads to agenesis of corneal endothelium with associated loss of anterior chamber, thinning of corneal stroma, features that are similar to RA signaling blocking mutants. Therefore, it is our interest to determine not only whether RA and TGF-β2 signaling act in concert or independently in CEC-like cells induction but also the underlying mechanism(s) in the future. We also observed in our preliminary experiments that pre-coating of laminin and chondroitin-6 sulphate was also necessary for successful CEC-like cell induction. It will be important to identify all signalling molecules and determine their potential relationship to RA and TGF-β2 signaling to improve the inductivity in the future.

Expression of N-cadherin coincides with the formation of corneal endothelium during eye development [22]. However, N-cadherin can not be used as a specific marker for corneal endothelium differentiation because of its wide distribution in many kinds of cells. Since there is no specific marker for CEC at present, several CEC differentiation and function-related proteins and transcription factors had to be taken into consideration together to identify the induction of CEC-like cells from NCC in this study. Positive expression of N-cadherin, FoxC1, Pitx2, Na+/K+ ATPase, ZO-1 as well as the following animal transplantation together proved that the cells induced from NCC had properties similar to those of native CEC. It is worth mentioning that our result showed that the staining of N-cadherin was in the cytoplasm of premature CEC-like cells and this pattern is different from that in other cells such as mature CEC, osteoblasts and many kinds of tumor cells, in which N-cadherin is restricted to the cell membrane. A similar phenomenon was reported in resent research on premature osteoblasts [28]. The mechanism of such different expression pattern of N-cadherin between premature and mature cells is not clear. According to previous findings [29], we assume that N-cadherin might be more related to cell differentiation and shaping in premature CEC, rather than cell adhesion and connection as it is in other mature cells. Mutations of FoxC1 and Pitx2 in mice were found to cause congenital defects in anterior segment development, including failure of the cornea to separate from the lens and failure of corneal endothelial differentiation. In this study, expression of FoxC1 sharply increased just before the appearance of polygonal cells and quickly fell after that. Unlike FoxC1, expression of Pitx2 was at a quite steady level at all the time points checked. The precise function of these two transcription factors is currently unknown. Our results indicated that FoxC1 might have a triggering effect on CEC differentiation, and Pitx2 might play a different role in corneal endothelial cell differentiation.

In conclusion, P75 and HNK-1 markers are both expressed by in vitro cultured rat NCC separated from cranial segment of the neural tube at embryonic day 9. CM from CEC could induce these NCC to differentiate into functional CEC-like cells. And transplantation of NCC-derived CEC-like cells into the anterior chamber together with short-term maintenance of eye-down position might have potential use in corneal endothelium deficiency management in the future.

## Materials and Methods

### Ethics Statement

This research was approved by experimentation committee of Shandong University Qilu Hospital, and all animals were treated according to the national and international rules of animal welfare, including the ARVO Statement for the Use of Animals in Ophthalmic and Vision Research. Design of the experiment and animal treatment were reported in detail during the ethics meeting, the proposal was subjected to defense and finally was passed by every member of the committee by voting.

### Cell Culture

SD rat NCC were cultured according to the method described by Sieber-Blum and Chokshi [30]. Briefly, Rat embryos were removed at embryonic day 9 and washed with D-Hank’s solution containing 100 U/ml penicillin and 100 U/ml streptomycin. Then they were placed in a petri dish and the neural tubes were dissected at the midbrain level (first 10 somites) using tungsten needles. The cranial segments were then transferred to a new petri dish coated with 25 ug/ml fibronectin and allowed to adhere for 15–20 min. Dishes were then flooded with NCC culture medium and incubated at 37°C in 5% CO_2_. Forty-eight hours later, the cranial segments were removed and the NCC that migrated out of the explants were harvested by trypsinization (0.05% trypsin/EDTA solution for 1 min). Cells were pelleted, resuspended and cultured for about seven days to reach confluence for the following experiments.

SD rat CEC were cultured according to the method previously described [31]. Briefly, Rat eyes were obtained after the end of other researcher’s research that did not involve any eye manipulations within 4 hours after death. The corneas were incubated in growth medium at 37°C overnight. After centrifugation, they were incubated in 0.02% EDTA for 1 h, and the loosened endothelial cells were detached from Descemet’s membrane by pipeting the corneas several times through a pipet. Endothelial cells were then centrifuged, resuspended in fresh DMEM/F12 medium containing 10% fetal bovine serum and cultured at 37°C with 5% CO_2_.

### CM Collection and NCC Induction

CM was derived by collecting the medium from cultured rat CEC at 70–90% confluence every 12 h. The collected medium was filtered (0.22 µm) to remove dead cells and stored at −80°C to preserve its biological activity.

CM was mixed with DMEM/F12 at a ratio of 3∶1 with 10% fetal bovine serum (FBS) as the differentiation medium. NCCs were cultured with the differentiation medium in 6-well plates coated with 10 µg/ml laminin and 10 mg/ml chondroitin-6 sulphate. After 20 days induction, when the cells remaining fibroblasts detached from the substrate, the polygonal CEC-like cells were thoroughly washed with warm phosphate buffer solution (PBS) and harvested for the following experiments.

### Immunofluorescence and Flow Cytometry

Primary antibodies were rabbit anti-P75 (Beijing Zhongshan, 1∶100), mouse anti-HNK-1 (Wuhan Boster, 1∶100), rabbit anti-N-cadherin (BD Biosciences, 1∶500), rabbit anti-zonula occludens-1 (Zymed laboratory, Invitrogen, 1∶100) and mouse anti-Na^+^/K^+^ ATPase(Santa Cruz, 1∶50). Goat anti-rabbit IgG antibodies conjugated with Rhodanmin (Beijing Zhongshan, 1∶100), goat anti-mouse IgG antibodies conjugated with FITC (Beijing Zhongshan, 1∶100), goat anti-rabbit IgG antibodies conjugated with FITC (Beijing Zhongshan 1∶100) and goat anti-mouse IgG antibodies conjugated with Rhodanmin (Beijing Zhongshan, 1∶100) were used as secondary antibodies. Cells were fixed in acetone for 10 mins at room temperature and incubated with antibodies diluted in phosphate buffered saline containing goat serum albumin at 4°C overnight (primary antibodies), or 37°C 30 mins (secondary antibodies). In negative controls, primary antibodies were substituted by phosphate buffer solution. Cell nuclei were counterstained with DAPI. Fluorescence was observed by using an Olympus inverted microscope.

For flow cytometric analysis of N-cadherin, cells were fixed and permeabilized with Cytofix/Cytoperm Kit (BD Biosciences Pharmingen) following manufacturer’s instruction. Cells were incubated with the primary antibody for N-cadherin (BD Biosciences) at 37°C for 20 mins, and then washed 3 times with phosphate buffer solution. Goat anti-rabbit IgG antibodies conjugated with FITC were used as secondary antibodies. In negative controls, primary antibodies were substituted by PBS. Fluorescence signals were collected by flow cytometry (BD Biosciences).

### Contact-inhibition Observation of CEC-like Cells

CEC-like cells were seeded on the surface of acellular porcine corneal matrix (APCM) scaffolds with a density of 3000 cells/mm^2^ and cultured under normal condition for a period of 30 days. Then half of the reconstructed corneal endothelium was double-stained with alizarin red S and trypan blue to show cell borders. The other half was fixed and 5 µm sections were subjected to HE staining. Photos were taken with an Olympus microscope.

### RNA Isolation and Real-time RT-PCR

FoxC1 and Pitx2 mRNA were detected by real time RT-PCR. The primer sequences were designed by Primer Premier 5 as follows: FoxC1, forward5′-CGGATCGGCTTAAACAACTCT-3′, reverse5′-GTTCCATTCCGTTTGGCTCT-3′; Pitx2, forward5′-GTCCAGCCCTGAAGTCGCAGAGA, reverse5′-AAAGTGAGTCCTCTGCCGGCG-3′. Specific primers for the β-actin gene were used as controls. Annealing temperature and cycles for FoxC1 were optimized as 62°C and 50 cycles, and for Pitx2 were 66°C and 50 cycles.

### Cryo-injury and Labeled CEC-like Cells Injection into the Anterior Chamber

The SD rat model of corneal endothelium deficiency was created according to a previous method [32], [33]. Briefly, SD rats weighing 200–250 g were anesthetized with intraperitoneal injection of 10% chloral hydrate (0.3 ml/100 g). The right eye was treated by immediately placing a brass dowel that had been cooled in liquid nitrogen on the corneal epithelium surface for 15 seconds. This procedure was repeated twice, each time allowing the eyeball to thaw by saline irrigation prior to refreezing. Then the anterior chamber was washed carefully three times with saline through a 0.5 mm width paracentesis to wash out the cell debris. Then CEC-like cells were transplanted following the method described by Mimura et al [34]. Briefly, CFDA SE-labeled 8.5×10^4^ CEC-like cells (for a rat cornea of 6 mm in diameter corresponding to a seeding density 3000 cells/mm^2^) were injected into the anterior chamber of each right eye, and cryo-injury alone group served as the control. Both experimental and control groups of rats were kept in the eye-down position for 24 hours under deep anesthesia.

Each surgical eye (n = 15) was photographed with a slit lamp microscope (Zeiss, Germany) 1, 3, 5, 7, 14, 21 and 28 days after surgery. Central corneal thickness was measured 1, 3, 5, 7, 14, 21 and 28 days after surgery. Images of CEC-like cells were taken and analyzed by confocal microscopy through focusing (HRT-II, Heidelberg Engineering, Germany). An average of readings was taken and results were reported as average value ±SD. SPSS 17.0 was used and comparisons with the control group (corneal endothelium deficiency without CEC-like cells transplantation, n = 15) were made using Student’s t-test. P<0.05 was considered significant.

### Histological Analysis

Postoperative rat eyes were removed and the corneas were viewed as whole-mounts under a fluorescence microscope to examine CFDA SE fluorescence. Then the corneas were fixed in formaldehyde and processed for paraffin embedding. Sections were subjected to HE staining and observed under a light microscope. Normal rat corneas served as controls.
