# Correction: Derivation of Corneal Endothelial Cell-Like Cells from Rat Neural Crest Cells In Vitro

**DOI:** 10.1371/annotation/931e92e2-ee2d-4f4f-b0b6-89a7a3f8fbad

**Published:** 2012-10-11

**Authors:** Chengqun Ju, Kai Zhang, Xinyi Wu

The following information was missing from the Funding section: This work was also supported by The National Natural Science Foundation of China (Grant Number: 81271716). 

